# Robust shrinking ellipsoid model predictive control for linear parameter varying system

**DOI:** 10.1371/journal.pone.0178625

**Published:** 2017-06-02

**Authors:** Zhang Longge, Yan Yan

**Affiliations:** Department of Mathematics and Physics, North China Electric Power University, Baoding, PR China; Chongqing University, CHINA

## Abstract

In this paper, a new off-line model predictive control strategy is presented for a kind of linear parameter varying system with polytopic uncertainty. A nest of shrinking ellipsoids is constructed by solving linear matrix inequality. By splitting the objective function into two parts, the proposed strategy moves most computations off-line. The on-line computation is only calculating the current control to assure the system shrinking into the smaller ellipsoid. With the proposed formulation, the stability of the closed system is proved, followed with two numerical examples to demonstrate the proposed method’s effectiveness in the end.

## 1. Introduction

Model predictive control (MPC), also known as receding or moving horizon control, is an effective control algorithm widely adopted in industry to deal with multivariable constrained control problem. MPC solves the constrained optimization problem at each sampling time and implements only the first element of the optimal control profile [1–6]. In recent ten years, some important branches is extended such as distributed MPC[7, 8], economic MPC[9, 10] and tube based MPC[11, 12] and so on.

Linear parameter varying (LPV) systems becomes a standard formalism in systems and control. It is introduced by Shamma [13] and it is an intermediate step between linear time-invariant (LTI) systems and non-linear plants. LPV systems can approximate many nonlinear systems and the gains can be automatically scheduled with respect to the parameters[14]. As the importance of LPV system, it has been widely investigated. Its common theme is to make the controller parameter dependent so that when the time-varying parameters are measured in real-time, the controller becomes self-scheduling and offers potential performance improvements over a fixed robust controller. Some researchers are concerned with the reducing the conservatism and improving the system’s performance. It is proposed that a state feedback MPC scheme based on a quasi-min-max algorithm [15], the first stage cost can be computed without any uncertainty, thus the first state cost can be determined separately from the rest of parameter changes. A robust dynamic output feedback MPC strategy is designed for a linear fractional representation represented systems[16]. Through the off-line designing robust state observer and on-line robust output feedback MPC controller designing, it is proposed a robust MPC scheme for LPV systems[17]. When the parameters have stochastic nature, via scenario optimization, a stochastic MPC for LPV systems is proposed[18]. In some practical applications, the varying parameters change rates are limited, and a novel algorithm is presented to estimate the future parameter variations which are predicted as a sequence of polytopic families [19, 20]. The designed feedback Robust MPC can improve the control performance. A robust MPC design method is proposed by Cao and Lin[21] to solve the influence of the actuator saturation, which degrades system performance and destroys the system’s stability. This method is improved by placing heavier weighting on the system corresponding to the actual linear feedback law[22].

On the other hand, the requirement of optimality leads to high on-line computation and this limits its application to relatively slow dynamics or small-scale process. To overcome this problem, some authors have proposed the off-line MPC. For example, a series of controllers corresponding to a sequence of nested asymptotically stable invariant ellipsoids is constructed off-line one with another[23]. This result is improved based on the nominal performance and followed with the improvement of the closed loop system’s feasibility and optimality[24].

In the LPV framework, it is assumed that the parameter is measureable or non-measurable. In the former case, the aforementioned researches have little use of the measured parameter vector. To solve this problem, this article aims to provide a formulation of decreasing the computation of the robust MPC for LPV system. The proposed method makes good use the information of the current states and parameter vector, and the future states can be described by the polytopic uncertain. The designed formulation employs the off-line designed shrinking ellipsoids which is first proposed in our previous work[25]. The shrinking ellipsoids $\left\{E_{i},i=1,2,\cdots N\right\}$ have the followed character: at the current time the state $x(k)\in E_{i}$, and at the next sampling time the state can be shrunk into the smaller ellipsoid with a controller, i.e. if $x(k)\in E_{i}$, then $x(k+1)\in E_{i+1}$. The on-line controller is calculated with low computation followed with the proof of the closed-loop system’s feasibility and stability.

The rest of this paper is organized as follows. Section 2 gives the problem description. In section 3, the shrinking ellipsoids are designed off-line, followed with the on-line control strategy. In section 4, two examples are presented to illustrate the effectiveness of the proposed strategy. Finally, we conclude the note in section 5.

The following notation will be used. Let *R*^*n*^ be the *n*-dimensional space of real valued vectors. For a matrix *Q* and a vector *x* ∈ *R*^*n*^, *x*^*T*^*Qx* will be denoted by $||x|{|}_{Q}^{2}$. The matrix inequality *A* > *B*(*A* ≥ *B*) means that *A* and *B* are square symmetric matrices and *A* − *B* is (semi-) positive definite. The measured or actual value of variable *x* at real time *k* will be denoted by *x*(*k*) (or *x*(*k* | *k*)). *x*(*k* + *i* | *k*), *i* ∈ {0, 1, 2, …} is the predicted value of *x* at a future prediction time *k* + *i* predicted at real time *k*. The identity matrix with proper dimension is denoted by *I*. * denotes the corresponding transpose of the lower block part of symmetric matrices.

## 2. Problem description

Consider a linear discrete-time LPV system whose system matrices are affine functions of a parameter vector *p*(*k*):

$$\begin{array}{l} x(k+1)=A(p(k))x(k)+B(p(k))u(k)\\ y(k)=Cx(k) \end{array}$$ (1)

where $[A(p(k)),B(p(k))]=\sum_{j=1}^{L}p_{j}(k)[A_{j},B_{j}]$, $x(k)\in R^{n_{x}}$ and $u(k)\in R^{n_{u}}$ denote the state and input respectively. The time-varying parameter vector *p*(*k*) = [*p*_1_(*k*), *p*_2_(*k*), ⋯, *p*_*L*_(*k*) ∈ *R*^*L*^] belongs to a convex polytope P, i.e., $\sum_{j=1}^{L}p_{j}(k)=1,\;0\leq p_{j}(k)\leq 1$.

The aim of the research is to find a feedback control law

u(k)=F(k)x(k) (2)

to achieve the followed performance cost

$$\begin{array}{l} \underset{u\left(k\right)}{\text{min}}\underset{[A(p(k)),B(p(k))]\in \Omega }{\text{max}}\;J_{\infty }(k)\\ J_{\infty }(k)=\sum_{i=0}^{\infty }\left\{x{\left(k+i|k\right)}^{T}Qx(k+i|k)+u{\left(k+i|k\right)}^{T}Ru(k+i|k)\right\} \end{array}$$ (3)

subject to state and output constraints

$$\begin{array}{cc} ||y(k+i|k)||\leq y_{\text{max }} & \forall k\geq 0,\forall i\geq 0 \end{array}$$ (4)

$$\begin{array}{cc} ||u(k+i|k)||\leq u_{\text{max }} & \forall k\geq 0,\forall i\geq 0 \end{array}$$ (5)

where *Q*, *R* > 0 are weighting matrices. The MPC for the LPV system is transformed into a convex optimization problem using parameter dependent Lyapunov function[26]. It is less conservative as compared with the result of M.V. Kothare for existing the loosen variables *G*. However, the convex optimization involves *L*^2^ + 3*L* LMIs (*L* is the number of convexes polytope) and it requires prohibitive on-line computation.

Lemma 1[26]. Consider the System (1) at the sampling time *k* with unknown parameters. Given *x*(*k* | *k*), *y*_max_, *u*_max_ a state feedback control law $u(k+i|k)=\sum_{j=1}^{L}\left[p_{j}\left(k\right)F_{j}(k)\right]x(k+i|k),\;F_{j}=Y_{j}G_{j}{}^{-1}$ is obtained by solving the following problem:

$$\underset{\gamma ,Y_{j},G_{j},Q_{j}}{\text{min}}\gamma$$ (6)

s.t.

$$\begin{array}{cc} \left[\begin{array}{cc} 1 & *\\ x(k|k) & Q_{j} \end{array}\right]\geq 0, & \forall j=1,2,\cdots L \end{array}$$ (7)

$$\begin{array}{ccc} \left[\begin{array}{cccc} G_{j}+G_{j}^{T}-Q_{j} & * & * & *\\ A_{j}G_{j}+B_{j}Y_{j} & Q_{l} & * & *\\ Q^{1/2}G_{j} & 0 & \gamma I & *\\ R^{1/2}Y_{j} & 0 & 0 & \gamma I \end{array}\right]>0, & \forall j=1,2,\cdots L, & \forall l=1,2,\cdots L \end{array}$$ (8)

$$\begin{array}{cc} \left[\begin{array}{cc} u_{\text{max}}^{2}I & *\\ Y_{}^{T} & G_{j}+G_{j}^{T}-Q_{j} \end{array}\right]\geq 0, & \forall j=1,2,\cdots L \end{array}$$ (9)

$$\begin{array}{cc} \left[\begin{array}{cc} y_{\text{max}}^{2}I & *\\ {\left(A_{j}G_{j}+B_{j}Y\right)}^{T}C^{T} & G_{j}+G_{j}^{T}-Q_{j} \end{array}\right]\geq 0, & \forall j=1,2,\cdots L \end{array}$$ (10)

On the other hand, the speed of the closed-loop response can be influenced by specifying a minimum decay rate on the state *x*(||*x*(*k*)|| ≤ *ρ*^*k*^ ||*x*(0)||, 0 < *ρ* < 1) as follows:

$$x{\left(k+i+1|k\right)}^{T}Q^{-1}x(k+i+1|k)\leq \rho^{2}x{\left(k+i|k\right)}^{T}Q^{-1}x(k+i|k)$$ (11)

for any [A(*k* + *i*), *B*(*k* + *i*)] ∈ Ω.

Lemma 2 [3]: For System (1), if it has a minimum decay rate of Eq (11), the following LMI must be satisfied:

$$\begin{array}{ccc} \left[\begin{array}{cc} \rho^{2}Q_{l} & *\\ A_{j}Q_{l}+B_{j}Y_{j} & Q_{l} \end{array}\right]\geq 0, & \forall j=1,2,\cdots L, & \forall l=1,2,\cdots L \end{array}$$ (12)

## 3. Shrinking ellipsoidal MPC for LPV system

In this section, it is assumed that both the parameter vector *p*(*k*) and the state *x*(*k*) are available in real-time. At sampling time *k* the system parameter vector *p*(*k*) is known exactly but unknown in the future. The designed strategy includes three stages: first, the method of seeking the minimum decay rate is presented for a fixed state; then a consequence of nested ellipsoids is constructed off-line based on the iterative method; finally the MPC algorithm is formulated. In the proposed method, most of the computation is moved off-line and only the low computation of calculating the input is left online.

Algorithm 3 (seeking the minimum decay rate). Consider the uncertain System (1) with Constraints (4) and (5). Let *ρ* = 1.

1. Investigate the feasibility of the following problem:
  Problem 1: $\underset{\gamma }{feasp}$ subject to Eqs (7) ~ (10) and (12).
2. If problem 1 is feasible, let *ρ* ≔ *ρ* − 0.01, go to step 1. Otherwise, return *ρ*.

Remark 4: The system is supposed to converge to the smaller invariant ellipsoid. The computed minimum decay rate is designed off-line and it is used to assure the stability of the closed-loop system.

Lemma 5 [25]. Suppose the matrices satisfy the followed condition 0 < *ρ*^2^*Q*_*i*_ < *Q*_*i*+1_ < *Q*_*i*_(0 < *ρ* < 1), define $E_{i}=\{x\in R^{n}|x^{T}Q_{i}^{-1}x\leq 1\}$ and $E_{i,\rho }=\{x\in R^{n}|x^{T}Q_{i}^{-1}x\leq \rho^{2}\}$, then $E_{i,\rho }\subset E_{i+1}\subset E_{i}$.

Algorithm 6 (off-line robust MPC). Consider the uncertain System (1) with Constraints (4) and (5). Given an initial feasible state *x*_1_, compute a sequence of minimizers as follows off-line. Let *i* ≔ 1.

1. Compute the minimum decay rate *ρ*_*i*_ at *x*_*i*_ using algorithm 3.
2. Compute the optimizer *γ*_*i*,*j*_, *Q*_*i*,*j*_, *X*_*i*,*j*_, *Y*_*i*,*j*_, *Z*_*i*,*j*_ at *x*_*i*_ using lemma 1 with an additional constraint $\rho_{i-1}^{2}Q_{i-1,j}\leq Q_{i,j}<Q_{i-1,j}$ (ignored at time *i* = 1, *j* = 1, 2⋯, *L*), store *γ*_*i*,*j*_, *γ*_*i*_, *Q*_*i*,*j*_ and $F_{i,j}(=Y_{i,j}Q_{i,j}^{-1})$, where $\gamma_{i}=\underset{j}{\text{max}}\left\{\gamma_{i,j}\right\}$
3. if *i* < *N*, choose a state *x*_*i*+1_ satisfying $x_{i+1}^{T}Q_{i,j}^{-1}x_{i+1}=\rho^{2}x_{i}^{T}Q_{i,j}^{-1}x_{i}$. Let *i* ≔ *i* + 1, go to step 1.

The state of the system is controlled into the smaller ellipsoid on-line. Suppose the state satisfying $x(k)\in E_{i},\;x(k)\notin E_{i+1}$, we select the proper controller to drive the system into $E_{i+1}$, that is $x(k+1)\in E_{i+1}$. Consider the following objective function which is split into two parts:

$$\begin{array}{ll} J_{0}^{\infty }(k)= & \underset{j_{0}^{0}}{\underset{︸}{x{\left(k\right)}^{T}Qx(k)+u{\left(k\right)}^{T}Ru(k)}}\\ & \;+\underset{j_{1}^{\infty }}{\underset{︸}{\sum_{i=1}^{\infty }x{\left(k+i|k\right)}^{T}Qx(k+i|k)+u{\left(k+i|k\right)}^{T}Ru(k+i|k)}} \end{array}$$ (13)

As the current state and parameter are unknown, so the first stage cost can be computed without uncertainty. This is the reason that the first control input can be separately from the rest of the future control law. Our on-line control strategy is based on the following two facts: the current state is in the ellipsoid $E_{i}$, but not in $E_{i+1}$, but in the following sampling time *k* + 1, the state is in $E_{i+1}$, that is $x(k+1|k)\in E_{i+1}$; *x*(*k* + 1 | *k*) and *p*(*k* + 1 | *k*) cannot be measured on-line, so in the future sampling time, the system is described by polytope uncertainty, and the future control law can be designed off-line using the Algorithm 6. So in this case $J_{1}^{\infty }\leq \gamma_{i+1}$, then the objective function satisfied

$$J_{\infty }(k)\leq x{\left(k|k\right)}^{T}Qx(k|k)+u{\left(k|k\right)}^{T}Ru(k|k)+\gamma_{i+1}$$ (14)

$x(k+1|k)\in E_{i+1}$:

$$\begin{array}{cc} x{\left(k+1|k\right)}^{T}Q_{i+1,j}^{-1}x(k+1|k)<1 & j=1,2,\cdots ,L \end{array}$$ (15)

The parameter vector *p*(*k*) and the current state *x*(*k* | *k*) are known, and only the input is free variable. The on-line work is only to compute the controller at the current sampling time

$$\underset{u(k),\upsilon }{\text{min}}\upsilon$$ (16)

satisfied Eq (15) and

$$x{\left(k|k\right)}^{T}Qx(k|k)+u{\left(k|k\right)}^{T}Ru(k|k)+\gamma_{i+1}<\upsilon$$ (17)

The Conditions (15) and (17) are equivalent to the followed LMIs respectively

$$\begin{array}{cc} \left[\begin{array}{cc} 1 & *\\ A(p(k))x(k)+B(p(k))u(k) & Q_{i+1,j} \end{array}\right]\geq 0 & j=1,2,\cdots ,L \end{array}$$ (18)

$$\left[\begin{array}{ccc} 1 & * & *\\ Q^{1/2}x(k|k) & (\upsilon -\gamma_{i+1})I & *\\ R^{1/2}u(k|k) & 0 & (\upsilon -\gamma_{i+1})I \end{array}\right]\geq 0$$ (19)

Theorem 7. The above optimization problem with the control law given by

$$\begin{array}{cc} U_{0}^{\infty }=[u(k|k),U_{1}^{\infty }], & U_{1}^{\infty }:\{u(k+i|k)=F(k+i|k)x(k+i|k),i\geq 1\} \end{array}$$ (20)

can be solved by the following semi-definite programming

$$\underset{u(k),\upsilon }{\text{min}}\upsilon$$ (21)

subject to Eqs (18) and (19).

Proof: Minimization of *x*(*k* | *k*)^*T*^ *Qx*(*k* | *k*) + *u*(*k* | *k*)^*T*^ *Ru*(*k* | *k*) + *γ*_*i*+1_ is equivalent to

$$\underset{u(k),\upsilon }{\text{min}}\upsilon$$

subject to *x*(*k* | *k*)^*T*^ *Qx*(*k* | *k*) + *u*(*k* | *k*)^*T*^ *Ru*(*k* | *k*) + *γ*_*i*+1_ ≤ *υ*, using Schur complements, it is equivalent to

$$\left[\begin{array}{ccc} 1 & * & *\\ Q^{1/2}x(k|k) & (\upsilon -\gamma_{i+1})I & *\\ R^{1/2}u(k|k) & 0 & (\upsilon -\gamma_{i+1})I \end{array}\right]\geq 0$$

which proves Eq (19);

Substituting Eq (1) into Eq (15), one can get

$$\begin{array}{cc} {\left[A(p(k))x(k)+B(p(k))u(k)\right]}^{T}Q_{i+1,j}^{-1}[A(p(k))x(k)+B(p(k))u(k)]<1 & j=1,2,\cdots ,L \end{array}$$

Using Schur complements it can be expressed as

$$\begin{array}{cc} \left[\begin{array}{cc} 1 & *\\ A(p(k))x(k)+B(p(k))u(k) & Q_{i+1,j} \end{array}\right]\geq 0 & j=1,2,\cdots ,L \end{array}$$

Remark 5: As the current parameter vector *p*(*k*) is known, there is no need to transform Eq (18) into the LMIs based on the convex of polytope.

The designed controller Eq (20) is composed of two parts: *u*(*k* | *k*) is the controller at the current sampling time and it can drive the state into the inner ellipsoid; $U_{1}^{\infty }$ is the off-line designed controller will be acted on the system in the following sampling time, and it can shrink the state into the smaller ellipsoid. The following theorem proves the closed-loop system’s stability.

Theorem 8. Given a dynamic System (1) and an initial state *x*(0) satisfying $||x(0)|{|}_{Q_{1}^{-1}}^{2}\leq 1$, the controller Eq (20) robustly asymptotically stabilizes the closed-loop system.

Proof: For the off-line minimization at *x*_*i*_, *i* = 2, …,*N*, the additional constraint $\rho_{i-1}^{2}Q_{i-1,j}\leq Q_{i,j}<Q_{i-1,j}$ is equivalent to $Q_{i-1,j}^{-1}<Q_{i,j}^{-1}\leq Q_{i-1,j}^{-1}/\rho_{i-1}^{2}$. From lemma 5, it is known that the left part of the inequality implies that the constructed asymptotically stable invariant ellipsoid $E_{i,j}$ is inside $E_{i-1,j}$, i.e. $E_{i,j}\subset E_{i-1,j}$. So it is guaranteed that $||x|{|}_{Q_{i,j}^{-1}}^{2}$ is monotonic decreasing with respect to the index *i*. The right part of the inequality can assure the algorithm’s feasibility, i.e., there exists $E_{i,j}$ in the feasible regions. From theorem 7, the control law Eq (20) is guaranteed to drive the state into the next nested ellipsoid. Lastly, the smallest $E_{N,j}$ is guaranteed to keep the state within $E_{N,j}$ and converge it to the origin.

Remark 10. As we all know, the fastest interior point algorithms show *O(RS*^*3*^*)* growth in computation where *R* is the total row size of the LMI system and *S* is the total number of scalar decision variables[23, 24, 27]. For LMI optimization Problem (6) the parameter *S* is given by $1+\frac{1}{2}L(1+n_{u}^{2}+n_{u}+2n_{x}^{2}+2n_{x})$ and *R* is given by *L*(7*n*_*x*_ + 2*n*_*u*_ + 1) while for Problem (21) *S* is given by 2 and *R* is given by 1 + *n*_*x*_ + *n*_*u*_. So the proposed strategy can reduce the on-line computation dramatically.

## 4. Numerical example

In this section, we present two examples to illustrate the effectiveness of the designed LPV system’s robust shrinking ellipsoid model predictive controller. The simulation is carried on Lenovo computer, and its processor is Intel^®^ core^™^ i5-4590cpu@3.30GHz.

Example 1. Consider the following LPV system given by[17]

$$\begin{array}{l} x\left(k+1\right)=A\left(\alpha \left(k\right)\right)x\left(k\right)+Bu\left(k\right)\\ y\left(k\right)=Cx\left(k\right) \end{array}$$ (22)

Where $A\left(\alpha \left(k\right)\right)=\left[\begin{array}{cc} 0.872 & -0.0623\alpha \left(k\right)\\ 0.0935 & 0.997 \end{array}\right]$, $B=\left[\begin{array}{c} 0.00935\\ 0.000478 \end{array}\right]$, $C=\left[\begin{array}{cc} 0.333 & -1 \end{array}\right]$. The initial states of the System (22) is assumed as $x\left(0\right)={\left[\begin{array}{cc} 10 & 0 \end{array}\right]}^{T}$ and the uncertain parameter *α*(*k*) belong to the following regions: *α*(*k*) ∈ [1, 5]. Then $\left[A\left(p\left(k\right)\right),B\left(p\left(k\right)\right)\right]=\sum_{j=1}^{2}p_{j}\left(k\right)\left[A_{j},B_{j}\right]$, where $A_{1}=\left[\begin{array}{cc} 0.872 & -0.0623\\ 0.0935 & 0.997 \end{array}\right]$, $A_{2}=\left[\begin{array}{cc} 0.872 & -0.3115\\ 0.0935 & 0.997 \end{array}\right]$, $B_{1}=B_{2}=\left[\begin{array}{c} 0.0935\\ 0.00478 \end{array}\right]$. We select some points on the *x* -axis, and get their minimum decay rate using the Algorithm 3 shown in Table 1.

**Table 1:** Selected points and corresponding minimum decay of example 1.

point	(10,0)	(8,0)	(6.4,0)	(5.12,0)	(4.1,0)	(3.28,0)	(2.63,0)	(2.11,0)	(1.69,0)
minimum decay	0.06	0.05	0.04	0.03	0.02	0.02	0.01	0.01	0.01

**Fig 1:**
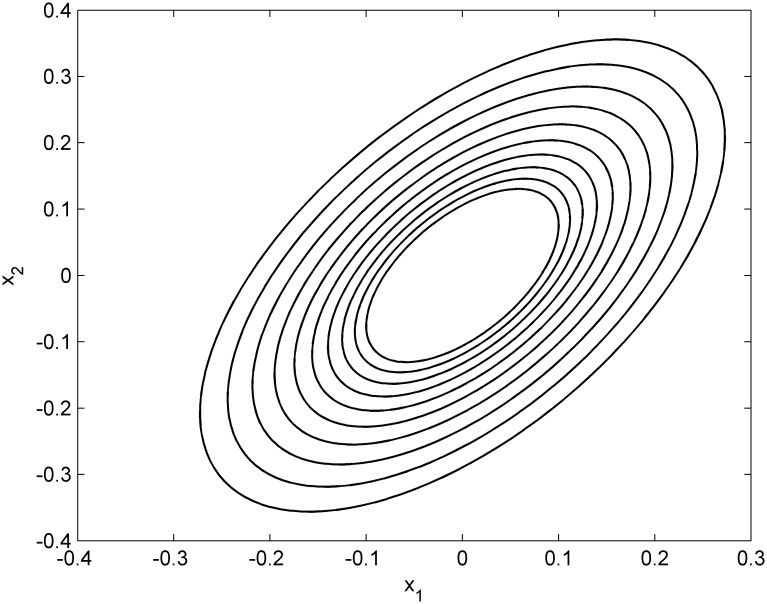
Nested ellipsoids of example 1.

**Fig 2:**
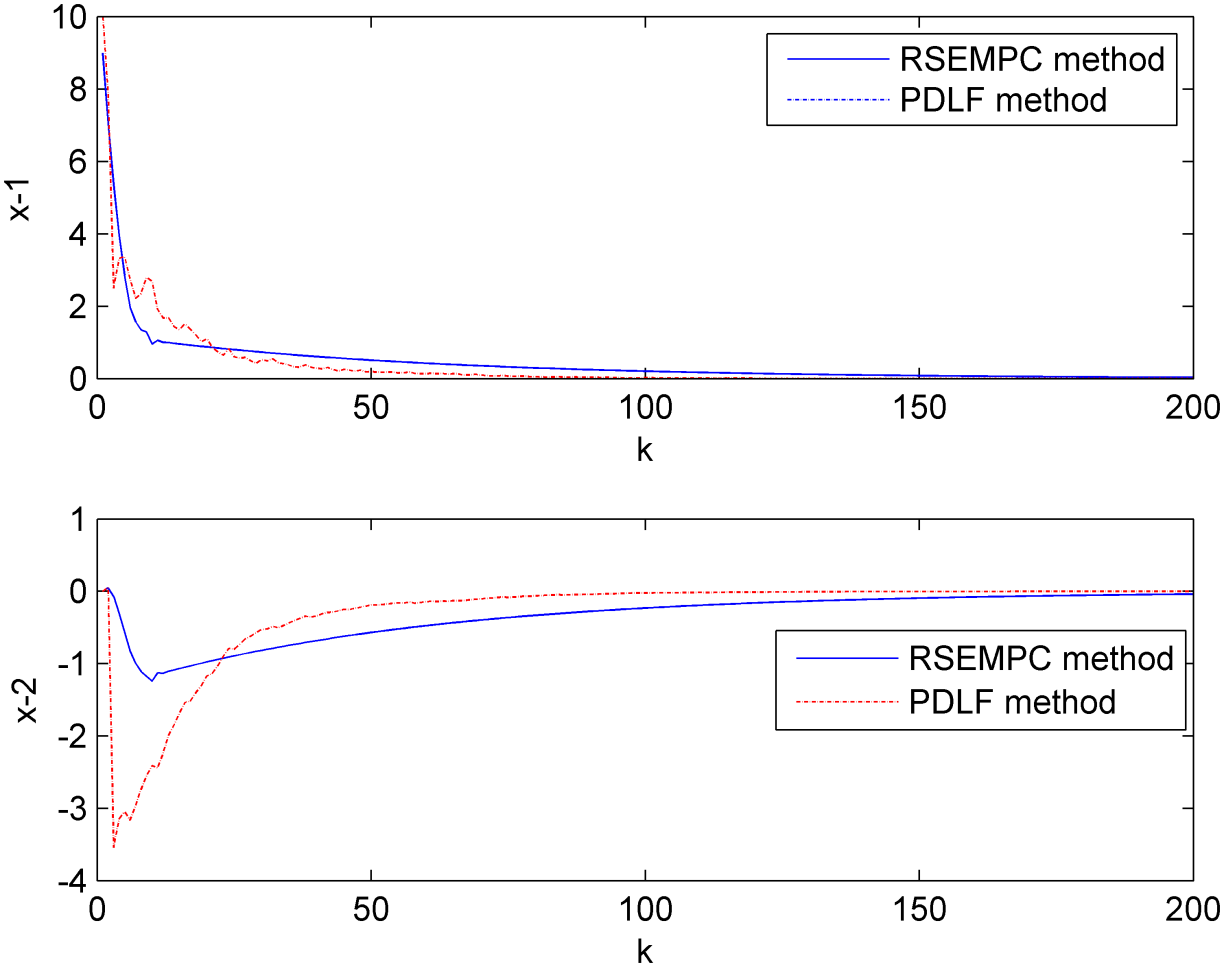
States of the closed-loop system of example 1.

It is shown that the minimum decay rate is smaller than 0.8 from Table 1. In the simulation, the decay rate is set to be 0.8 and the nested ellipsoids is shown in Fig 1. The states of the controlled closed-loop system using the designed method (RSEMPC method) compared with the method in paper[26](PDLF method) are shown in Fig 2. The runtime of RSEMPC method is 0.001062 second while the PDLF method is 6.410566 second.

It is shown that the computation time of the RSEMPC method is much smaller than the PDLF method and most of the computation is left offline.

Example 2. Consider the following uncertain nonlinear model for non-isothermal control of a continuously stirred tank reactor (CSTR) where the exothermic reaction *A* → *B* takes place. A cooling coil is used to remove heat that is released in the exothermic reaction. The reaction rate constant *k*_0_ and the heat of reaction Δ*H*_*r*×*n*_ are considered to be the uncertain parameters. The linearized model based on the component balance and the energy on the component balance is given as follows

$$\left[\begin{array}{c} {\dot{C}}_{A}\\ \dot{T} \end{array}\right]=\left[\begin{array}{cc} -\frac{F}{V}-k_{0}e^{-E/RT_{s}} & -\frac{E}{RT_{s}^{2}}k_{0}e^{-E/RT_{s}}C_{As}\\ -\frac{\Delta H_{r\times n}k_{0}e^{-E/RT_{s}}}{\rho C_{p}} & -\frac{F}{V}-\frac{UA}{V\rho C_{p}}-\Delta H_{r\times n}\frac{E}{\rho C_{p}RT_{s}^{2}}k_{0}e^{-E/RT_{s}}C_{As} \end{array}\right]\left[\begin{array}{c} C_{A}\\ T \end{array}\right]+\left[\begin{array}{c} 0\\ -2.098\times 10^{5}\frac{T_{s}-365}{V\rho C_{p}} \end{array}\right]\left[\begin{array}{c} C_{A,F}\\ F_{c} \end{array}\right]$$ (23)

where *C*_*A*_ denotes the concentration of *A* in the reactor, *T* denotes the reactor temperature and *T*_*C*_ denotes the temperature of coolant stream. The rate of reaction is first order with respect to component *A*. The relevant constants for the CSTR dynamic model are illustrated in Table 2.

**Table 2:** Relevant constants for the CSTR dynamic model.

*C*_*A*_	concentration of *A*
*F*	Feed flowrate
*V*	Volume of reactor
*C*_*A*,*F*_	Feedback concentration of *A*
*k*_0_	reaction rate constant
*E*	Activation energy
*R*	Universal gas constant
*T*	Reactor temperature
*T*_*s*_	Reactor temperature at steady state
*C*_*AS*_	concentration of *A* at steady state
*ρ*	Mean density of water
*C*_*p*_	Heat capacity
Δ*H*_*r*×*n*_	heat of reaction
*F*_*C*_	Coolant flow

**Fig 3:**
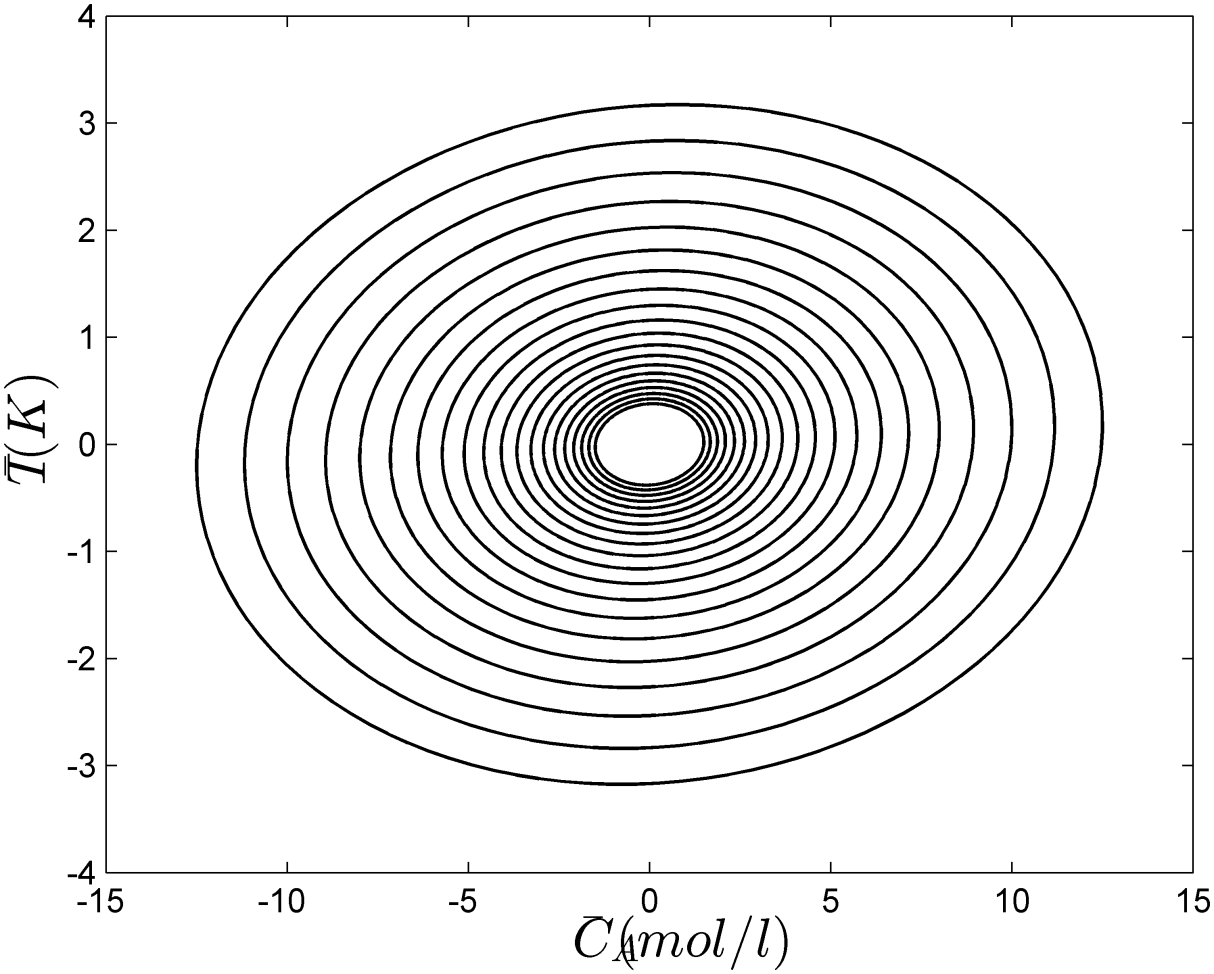
Nested ellipsoids of example 2.

**Fig 4:**
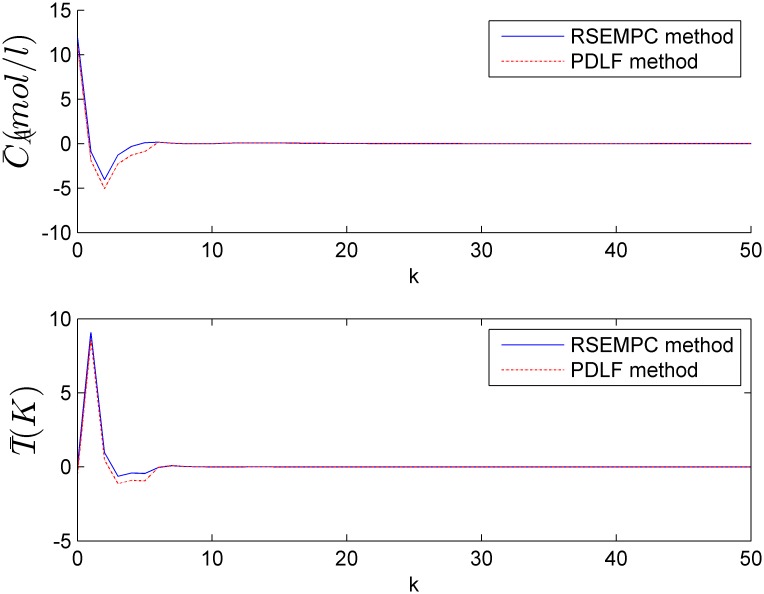
States of the closed-loop system of example 2.

Let ${\bar{C}}_{A}=C_{A}-C_{A,eq}$, $\bar{T}=T-T_{eq}$, ${\bar{C}}_{A,F}=C_{A,F}-C_{A,F,eq}$ and ${\bar{F}}_{C}=F_{C}-F_{C,eq}$, where the subscript *eq* is used to denote the corresponding variable at equilibrium condition. The discrete-time model is obtained by discretizing using Euler first-order approximation with a sampling time of 0.15 min (*F* = 1 *m*^3^ / min, *V* = 1 *m*^3^, *k*_0_ = 10^9^ ~ 5 × 10^9^ min^−1^, E / *R*= 8330.1*K*, −Δ*H*_*r*×*n*_ = 10^7^ ~ 5 × 10^7^ *cal* / *kmol*, *ρ* = 10^6^ *g* / *m*^3^, *UA* = 5.34 × 10^6^ *cal* / *K*, *C*_*p*_ = 1 *cal* / (gK), *T*_*s*_ = 394*K* and *C*_*AS*_ = 0.265 *kmol* / *m*^3^)

$$x\left(k+1\right)=\left[\begin{array}{cc} 0.85-0.0986\alpha \left(k\right) & -0.0014\alpha \left(k\right)\\ 0.9864\alpha \left(k\right)\beta \left(k\right) & 0.0487+0.01403\alpha \left(k\right)\beta \left(k\right) \end{array}\right]x\left(k\right)+\left[\begin{array}{c} 0\\ -0.912 \end{array}\right]u\left(k\right)$$ (24)

where $x\left(k\right)=\left[{\bar{C}}_{A}\left(k\right);\bar{T}\left(k\right)\right]$, $\;u\left(k\right)=\left[{\bar{C}}_{A,F}\left(k\right);{\bar{F}}_{C}\left(k\right)\right]$, 1 ≤ *α*(*k*) = *k*_0_ / 10^9^ ≤ 5 and 1 ≤ *β*(*k*) = −Δ*H*_*r*×*n*_ / 10^7^ ≤ 5. The polytopic Ω = *Co*{*A*_1_, *A*_2_, *A*_3_, *A*_4_}, $A_{1}=\left[\begin{array}{cc} 0.7514 & -0.0014\\ 0.9864 & 0.06273 \end{array}\right]$, $A_{2}=\left[\begin{array}{cc} 0.357 & -0.007\\ 4.932 & 0.11885 \end{array}\right]$, $A_{3}=\left[\begin{array}{cc} 0.7514 & -0.0014\\ 4.932 & 0.11885 \end{array}\right]$, $A_{4}=\left[\begin{array}{cc} 0.357 & -0.007\\ 24.66 & 0.39945 \end{array}\right]$. It is the same as example 1, we can find the minimum decay rate is smaller than 0.8. In the simulation, the decay rate is set to be 0.8 and the nested ellipsoids constructed off-line by the prosed algorithm is shown in Fig 3. If the state of the system is in one ellipsoid at the present time *k*, then at the next sampling time *k* + 1, the state must be in the adjacent interior ellipsoid, so we call them as the shrinking ellipsoids. Table 3 shows the overall on-line numerical burdens compared with PDLF method. It is shown that the designed algorithm requires smaller on-line computation. Fig 4 shows the closed-loop responses of the system.

**Table 3:** On-line numerical burdens in example 1.

Algorithm	CPU time(s) per step
RSEMPC	0.001
PDLF	0.291

## 5. Conclusions

In this paper, a novel off-line MPC synthesis approach for a LPV system is presented. A sequence of state feedback gains corresponding to the sequences of nested ellipsoids is pre-computed. The on-line computation is only to calculate the input to control the states into the inner ellipsoids and it only left the current controller as a free variable, so most of the computation is moved off-line. The effectiveness of the propose method is illustrated by two simulation examples.

## Supporting information

S1 Fig States and ellipsoids of example 1. (RAR)

S2 Fig States and ellipsoids of example 2. (RAR)
